# The Beneficial Roles of SIRT1 in Neuroinflammation-Related Diseases

**DOI:** 10.1155/2020/6782872

**Published:** 2020-09-14

**Authors:** Fangzhou Jiao, Zuojiong Gong

**Affiliations:** Department of Infectious Diseases, Renmin Hospital of Wuhan University, Wuhan, 430060 Hubei, China

## Abstract

Sirtuins are the class III of histone deacetylases whose deacetylate of histones is dependent on nicotinamide adenine dinucleotide (NAD+). Among seven sirtuins, SIRT1 plays a critical role in modulating a wide range of physiological processes, including apoptosis, DNA repair, inflammatory response, metabolism, cancer, and stress. Neuroinflammation is associated with many neurological diseases, including ischemic stroke, bacterial infections, traumatic brain injury, Alzheimer's disease (AD), and Parkinson's disease (PD). Recently, numerous studies indicate the protective effects of SIRT1 in neuroinflammation-related diseases. Here, we review the latest progress regarding the anti-inflammatory and neuroprotective effects of SIRT1. First, we introduce the structure, catalytic mechanism, and functions of SIRT1. Next, we discuss the molecular mechanisms of SIRT1 in the regulation of neuroinflammation. Finally, we analyze the mechanisms and effects of SIRT1 in several common neuroinflammation-associated diseases, such as cerebral ischemia, traumatic brain injury, spinal cord injury, AD, and PD. Taken together, this information implies that SIRT1 may serve as a promising therapeutic target for the treatment of neuroinflammation-associated disorders.

## 1. Introduction

Inflammation is a physiological response of the immune system to harmful infectious and noninfectious stimuli. In response to such stimuli, macrophages, immune cells, and vascular cells take concerted reactions to maintain or restore the integrity of tissue [1]. However, a sustained inflammatory response can be harmful to the body [2]. Inflammatory responses that are centralized in the central nervous system (CNS) are referred to as neuroinflammation [3]. Neuroinflammation has been implicated as the pathogenesis in multiple neurological diseases, including ischemic stroke, bacterial infections, traumatic brain injury, and neurodegenerative diseases such as Alzheimer's disease (AD), Parkinson's disease (PD), amyotrophic lateral sclerosis (ALS), multiple sclerosis (MS), age-related dementia, and Huntington's disease (HD) [4–6].

Due to the existence of the blood-brain barrier (BBB), the peripheral immune cells and molecules have difficulty entering the CNS. Hence, resident innate immune cells of the CNS need to deal directly with various toxins, pathogens, and tissue damage [1]. Pattern recognition receptors (PRRs) have an essential effect on the recognition of pathogen-associated molecular patterns (PAMPs) and derived danger-associated molecular patterns (DAMPs) during inflammatory conditions. There are four families of PRRs, namely, Toll-like receptors (TLRs), NOD-like receptors (NLRs), retinoid acid-inducible gene-1- (RIG-1-) like receptors (RLRs), and C-type lectin receptors (CLRs) [7]. In CNS, these PRRs are mainly expressed in microglia, astrocytes, endothelial cells, dendritic cells (DCs), and oligodendrocytes [8, 9]. There are several innate immune cells in the brain, including astrocytes, microglia, macrophages, natural killer (NK) cells, mast cells, and oligodendrocytes [10]. Among these cells, microglia are the primary innate immune cells, and they constitute 10-15% of all the glial cells. Under pathological conditions, microglia are activated by PAMPs and characterized by rapid proliferation and production of proinflammatory cytokines [11]. Acute neuroinflammation is a key response to clear pathogens and to repair tissue damage. However, if acute neuroinflammation remains unresolved, it can lead to chronic inflammation and neurodegeneration [12].

Sirtuins are the class III of histone deacetylases and are nicotinamide adenine dinucleotide- (NAD+-) dependent enzymes. The family consists of seven members in mammals, namely, silent information regulator 1 (SIRT1), SIRT2, SIRT3, SIRT4, SIRT5, SIRT6, and SIRT7 [13]. The sirtuins are epigenetic modulators involved in many physiologic activities, such as genomic stabilization, cancer, stress response [14], apoptosis, metabolism, senescence, proliferation [15], and inflammation [16]. Among all sirtuins, SIRT1 is the first sirtuin to be recognized and is well studied [17]. Many studies have indicated that SIRT1 could regulate the inflammation response in multiple tissues and cells [16]. An increasing number of studies have reported that SIRT1 was closely linked with neuroinflammation [18]. However, the mechanistic pathways involving SIRT1 and neuroinflammation remain poorly defined. Hence, the focus of this review is to summarize the latest advances regarding the potential beneficial roles of SIRT1 in the regulation of neuroinflammation. This study may serve as a useful foundation for the design of a drug for future neuroinflammation and neurodegenerative disease treatments.

## 2. SIRT1 Structure

**Figure 1:**
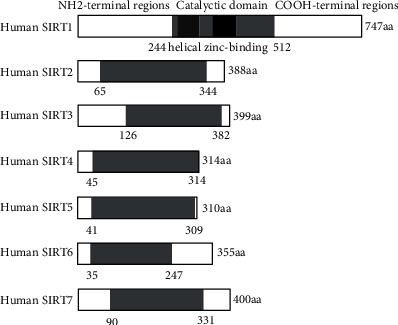


Sirtuins contain a conserved catalytic domain, NAD^+^ binding domains, and flanking variable NH2- and COOH-terminal regions [19, 20] (Figure 1). The diversity of sirtuin amino acid sequences contribute to their cellular localization, catalysis activity, and function. The human sirtuins are divided into four classes. SIRT1, SIRT2, and SIRT3 are class I showing close homology to yeast Sir2. SIRT4 is class II, and SIRT5 is class III. SIRT6 and SIRT7 belong to class IV sirtuins [21] Among sirtuins, SIRT1 has the largest extensions of terminal regions. The SIRT1 protein (747 amino acids) is made up of the conserved catalytic core (244–512 residues), the COOH-terminal region (1–180 residues), and the NH2-terminal region (513–747 residues) [22]. SIRT1 protein contains the nuclear localization signal (KRKKRK) in 41-46 residues [19]. Thus, it is easy to understand that SIRT1 is identified as a nuclear protein. However, numerous evidences indicated that SIRT1 is localized in both the nucleus and cytoplasm in several cells [23–25]. Under certain conditions, SIRT1 can shuttle between the nucleus and cytoplasm [26]. The nuclear import and export sequences in the NH2-terminal region of SIRT1 have been identified as a regulatory mechanism of nucleocytoplasmic shuttling of SIRT1[27]. Aside from SIRT1, the other six sirtuins have distinct subcellular localizations. SIRT2 is primarily located in the cytoplasm [28], but it can shuttle from the cytoplasm to the nucleus [29]. SIRT3, SIRT4, and SIRT5 are generally identified as mitochondrial proteins. Recent studies indicated that SIRT3 is not exclusive in mitochondria. SIRT3 primarily localizes in the nucleus and translates to the mitochondria after ultraviolet radiation or etoposide treatment [30]. Apart from SIRT1, SIRT6 and SIRT7 also show nuclear localization. SIRT6 is a chromatin-bound protein, whereas SIRT7 is concentrated in the nucleolus [31].

## 3. Catalytic Mechanism of SIRT1

**Figure 2:**
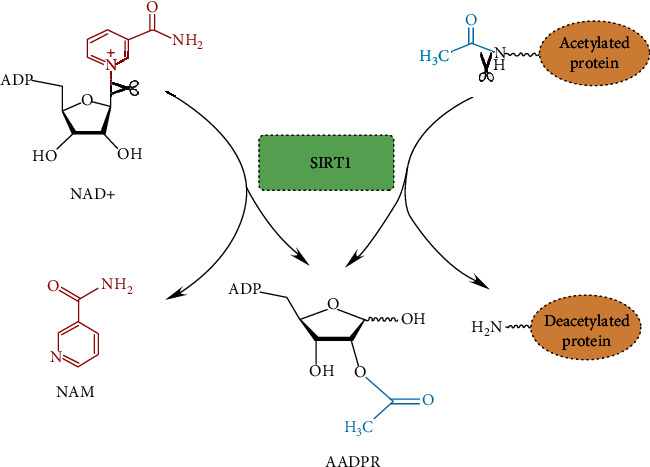


As the histone deacetylases, mammalian sirtuins share a conserved catalytic domain. For human SIRT1, the catalytic core consists of two domains. The larger NAD^+^-binding domain composes of a Rossmann fold, and the smaller domain consists of a helical (269–324 residues) and a zinc-binding module (362–419 residues) [32]. On the basis of the three-dimensional crystal structures of a sirtuin protein, the catalytic pocket between large and small domains is frequently divided into three active sites: the site A is involved in binding of the adenine-ribose part of NAD, the site B is involved in binding of the nicotinamide-ribose portion, and the site C is the deep location of the NAD-binding pocket [33]. The molecular mechanism of the deacetylation reaction is complicated. Several studies have reported the possible mechanism of the reaction. Briefly, the catalytic reaction begins with forming the ternary complex, which is mediated by NAD+, and an acetylated substrate both binds to the sirtuin enzyme [34]. In this deacetylation process, the glycosidic bond of NAD+-linking nicotinamide and ADP-ribose moiety is cleaved, and the free nicotinamide is released. Then, the acetyl moiety is transferred to ADP-ribose and produces acetyl-ADP-ribose (2′-O-acetyl-ADP-ribose, AADPR) and deacetylated protein [35] (Figure 2). Although human sirtuins belong to histone deacetylases, it should be pointed out that SIRT1, SIRT2, SIRT3, and SIRT7 display deacetylase activity, whereas the deacetylase activities of SIRT4–6 are considered to be weak. SIRT4 has ADP-ribosyltransferase and lipoamidase activity [36, 37]. SIRT5 possesses strong desuccinylation and depropanediylation activity [38]. SIRT6 was initially reported to have deacetylase activity on specific histone acetyl peptides H3 K9 and H3 K56 [39]. A recent study found that SIRT6 has demyristoylation activity by regulating the hydrolysis of fatty acylation of lysine residues [40].

## 4. SIRT1 Substrates and Function

SIRT1 is the most well-studied member of the sirtuin family. As a deacetylate histone, it is easy to understand that SIRT1 can regulate the deacetylation of the acetyl-lysine of histone substrate. A previous study confirmed that SIRT1 deacetylates histone H4 at lysine 16 (K16), histone H3 at lysine 9 (K9), and histone H1 at lysine 26 (K26) [41]. In addition to the deacetylate of histones, SIRT1 could catalyze the deacetylation of many nonhistone substrates. A growing number of evidence indicate that SIRT1 can deacetylate the lysine residues of a variety of substrates, such as p53, nuclear factor kappa B (NF-*κ*B), Ku70, FOXO1, FOXO3, FOXO4, hypoxia-inducible factor-1a (HIF-1*α*), HIF-2*α*, proliferator activated receptor *γ* coactivator 1*α* (PGC-1*α*), and signal transducer and activator of transcription 3 (STAT3) (Table 1). Thus, this wide range of targets reflects that SIRT1 has a strong capacity to regulate multiple physiological functions.

p53 is the first nonhistone target of SIRT1; deacetylation of p53 at lysine 382 (K382) results in the repression of p53-dependent apoptosis in H1299 cells in response to DNA damage [42, 43]. As a tumor suppressor, the functional and transcriptional activities of p53 are blocked by the deacetylation of SIRT1 [44]. Thus, SIRT1 provides a prosurvival response following DNA damage. NF-*κ*B is an important transcription factor that regulates the transcription of many genes. SIRT1 can deacetylate the p65 subunit of NF-*κ*B at lysine 310. It is interesting that deacetylation of p65 augments TNF*α*-induced apoptosis in non-small-cell lung cancer (NSCLC) cells. A possible hypothesis is the different apoptotic signaling and stimulations [45]. In addition to the apoptosis role, the deacetylation of p65 has been shown to regulate the inflammation response [46]. Ku70 is a DNA repair protein; SIRT1 can deacetylate and activate Ku70 by direct interaction. The deacetylation of Ku70 increases the DNA repair capacity [47, 48]. The Forkhead box O (FOXO) transcription factor family is a subclass of Forkhead transcription factors, including four members FOXO1, FOXO3, FOXO4, and FOXO6. Many evidences suggest that the deacetylation of FOXO1, FOXO3, and FOXO4 can be mediated by SIRT1 [49, 50]. For example, the activation of FOXO1 mediated by SIRT1 induces the apoptosis of prostate cancer cells [51]. Deacetylation of FOXO3 by SIRT1 potentiates the ability of FOXO3-induced cell cycle arrest and inhibits cell apoptosis mediated by FOXO3 in response to oxidative stress [52]. In addition, the hyperacetylation and transcriptional activity of FOXO4 can be mediated by peroxide stress, while deacetylation of FOXO4 by SIRT1 enhances the protective effect against oxidative stress [53]. Hypoxia-inducible factors are transcription factors that are critical in the regulation of metabolism under hypoxia. Previous studies have found that both HIF-1*α* and HIF-2*α* can be deacetylated by SIRT1 [54]. The deacetylation of HIF-1*α* by SIRT1 enhances the expression of HIF-1*α* target genes (VEGF, GLUT1, and MMP2) and then results in the promotion of cell invasion [55]. SIRT1 augments the expression of HIF-2*α* target gene erythropoietin (prosurvival factor) by deacetylation of HIF-2*α* [56]. PGC-1*α* is a transcriptional coactivator with multiple functions, including mitochondrial biogenesis and glucose and fatty acid metabolism [57]. Deacetylation of PGC-1*α* by SIRT1 enhances the effects of PGC-1*α* on fatty acid oxidation and gluconeogenesis genes in response to caloric restriction [58, 59]. STAT3 is a cellular signal transcription factor that mediates many cellular functions [60]. SIRT1-mediated deacetylation of STAT3 is involved in the regulation of gluconeogenic genes [61] and cell proliferation [62]. In addition to these highlighted substrates, numerous SIRT1 substrates have been discovered [63]. Taken together, these multiple substrates indicate that SIRT1 participates in the regulation of multiple physiological functions, including apoptosis, DNA repair, inflammatory response, metabolism, cancer, and stress.

## 5. SIRT1 Activator Resveratrol

Resveratrol (3,5,4′-trihydroxy-trans-stilbene) is a natural polyphenol found in a variety of plant species, which is a natural antibiotic compound synthesized by plants [64]. Numerous studies have shown that resveratrol has many functions, including antioxidant capacity, anti-inflammation, neuroprotection, and anticancer [65]. Many studies confirm that resveratrol is a natural activator of SIRT1. Previous experiments have shown that resveratrol doubled the catalytic rate of SIRT1 at about 11 *μ*M [66]. Dozens of studies report that the anti-inflammatory effects of resveratrol are dependent on SIRT1 [67–70]. However, recent evidence shows that resveratrol may activate SIRT1 activity indirectly. One mechanism in which resveratrol activates SIRT1 involves cAMP and AMPK [71]. Another mechanism is that resveratrol activates Lamin A, which is a protein activator of SIRT1 [72]. Several studies have shown that resveratrol regulated neuroinflammation through SIRT1. A recent study indicated that resveratrol inhibits neuroinflammatory response in aged rats after anesthesia and surgery by activation of SIRT1 [73]. Similarly, resveratrol inhibited the expressions of inflammatory factors in LPS-induced activation of microglia cells by upregulation of SIRT1 [74].

## 6. SIRT1 Distribution in CNS

Previous studies have confirmed the distribution of SIRT1 in a wide variety of mouse organs, including the brain, spinal cord, eyes, heart, liver, kidney, lung, and testis [75, 76]. It is also widely expressed in several human tissues and organ, including the brain [77], liver [78], heart [79], skeletal muscle [80], pancreas [81], and adipose tissue [82]. It is interesting that the high levels of SIRT1 expression are observed in the embryonic brain, spinal cord, and heart [75]. An anatomical study of the rodent and human nervous systems indicated that the distribution of SIRT1 is localized in the areas of the hippocampus, prefrontal cortex, and basal ganglia [83]. In addition to these regions, a mouse anatomical study revealed that SIRT1 is also expressed in the hypothalamus and cerebellum [84]. Moreover, this study also indicated that SIRT1 mRNA is mainly expressed in neurons [84]. Recent studies had reported that several cellular types (mouse astrocyte, microglia, and oligodendrocytes) express SIRT1 proteins [85, 86]. Taken together, these studies suggest that SIRT1 is widely expressed in CNS.

## 7. SIRT1 and Neuroinflammation

**Figure 3:**
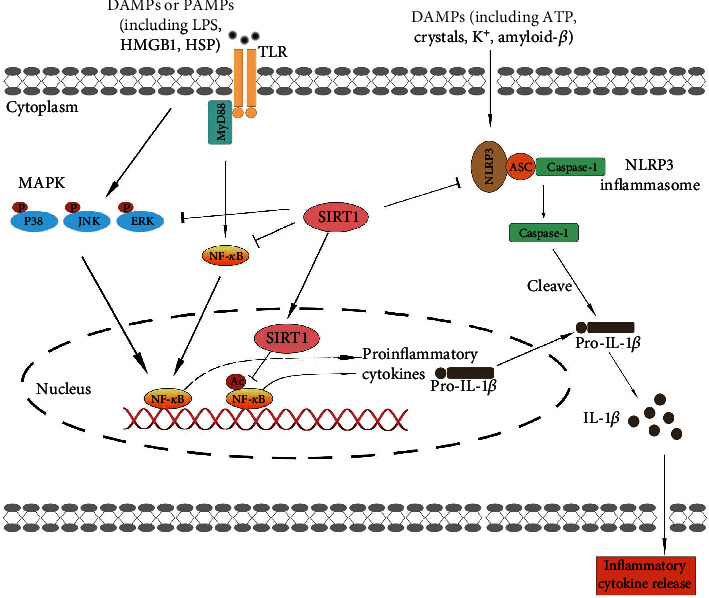


The innate immune system is the first line of defense against infection and injury and plays critical roles in the maintenance of brain homeostasis. It has been shown that astrocytes, microglia, macrophages, NK cells, mast cells, and oligodendrocytes serve as the innate immune cells of the brain [11]. Among these cells, microglia are mainly innate immune cells that are derived from the embryonic yolk sac [87]. Under physiological conditions, microglia constantly survey their microenvironments and present in the resting state with small cellular bodies and slender branching [88]. Under pathological conditions, microglia are activated and converted into amoeboid reactive cells, with the production of proinflammatory factors [89]. Overactivation of microglia has been implicated to the development of neuroinflammation [90]. In addition to microglia, astrocytes also are key innate immune cells involved in the regulation of neuroinflammation [91]. Astrocytes became reactive astrocytes with significant changes in morphology, function, and gene expression in response to CNS injuries and diseases [92]. A previous study suggests that the increase of inflammatory factor (TNF-*α*, IL-1*β*, and iNOS) gene expression is observed in both primary microglia and astrocytes after treatment with LPS [85]. Growing evidence supports a role for SIRT1 in the regulation of inflammatory responses in CNS. For example, the SIRT1 activator decreases the secretion of TNF-*α* and IL-6 in microglial BV2 cells by LPS stimulation, while the SIRT1 inhibitor increases the inflammatory factors release [93]. Another study found that SIRT1 inhibition upregulates the gene expression levels of TNF-*α* and IL-1*β* and ROS production in astrocytes after stimulation with LPS [94]. However, the molecular mechanisms involving SIRT1 and neuroinflammation are still unclear. Emerging potential regulatory mechanisms of SIRT1 in the process of neuroinflammation may involve the NF-*κ*B pathway, Toll-like receptor pathway, NLRP3 inflammasome pathway, and MAPK signal pathway (Figure 3). These signal pathways will be discussed in the following paragraphs.

### 7.1. SIRT1 and NF-*κ*B

The transcription factor NF-*κ*B family regulates the expression of multiple genes involved in the inflammatory process [95]. It has been shown that the NF-*κ*B family has five subunits: p65 (also RelA), p50, p52, c-Rel, and RelB. The relative pathway mediated by NF-*κ*B complex p65/p50 is commonly defined as the canonical NF-*κ*B pathway. Generally, NF-*κ*B complexes are sequestered by NF-*κ*B inhibitory proteins (I*κ*Bs) in the cytoplasm. Once the pathway is activated, the I*κ*B proteins are activated by phosphorylation and its subsequent degradation, resulting in the nuclear translocation of NF-*κ*B complexes and then regulation of target gene expression [96]. A growing number of studies have shown that SIRT1-mediated NF-*κ*B participates in the regulation of inflammation response and diseases. As described previously, SIRT1 has demonstrated the capacity of the deacetylating p65 subunit [45]. Thus, it is easy to understand that SIRT1 mediates the inflammation process by directly regulating the NF-*κ*B pathway. Bone marrow-derived macrophages (BMDMs) from myeloid cell-specific SIRT1 knockout mice result in the hyperacetylation of NF-*κ*B and the increase of proinflammatory cytokines [97]. Activation of SIRT1 by resveratrol inhibits TNF-*α*-induced inflammation responses in 3T3 fibroblasts by deacetylating the Lys310 residue of the p65 subunit [70]. Similarly, resveratrol decreases the TNF-*α*-mediated inflammation responses in human chondrocytes by deacetylating NF-*κ*B p65 [98].

A number of studies demonstrated that the SIRT1 and NF-*κ*B pathways are involved in neuroinflammation. Accumulating studies indicate that the decrease of SIRT1 and the activation of the NF-*κ*B pathway participate in the progress of neuroinflammation and diseases. A recent study found that microglial SIRT1 deficiency aggravates memory deficits and increases IL-1*β* transcription in aging mice [99]. High levels of acetylation of NF-*κ*B/RelA in primary cortical neurons following oxygen-glucose deprivation (OGD) have been found [100]. Moreover, several neural cell experiments have demonstrated that SIRT1 activator resveratrol suppresses inflammatory cytokines via inhibition of NF-*κ*B transcriptional activity [101, 102]. Furthermore, many animal models indicated that SIRT1 activator resveratrol inhibits the inflammatory cytokines and improves the survival of the neuronal cell by regulating the acetylation of NF-*κ*B p65 [73, 103–105] (Table 2). In addition to the direct regulation of the NF-*κ*B pathway by deacetylating the p65 subunit, some studies demonstrated that SIRT1 could indirectly regulate the NF-*κ*B pathway by other targets, such as PPAR*α* [46, 106]. Taken together, SIRT1 can suppress the neuroinflammation involved in regulating NF-*κ*B signaling by deacetylation of the p65 subunit and inhibition of NF-*κ*B transcriptional activity.

### 7.2. SIRT1 and Toll-Like Receptors

Several toll-like receptors (TLRs) are widely expressed in the immune system of the brain, including microglia, astrocytes, and neurons [6]. As membrane receptors, TLRs recognize several molecules, such as lipopolysaccharide (LPS), heat shock proteins, high-mobility group protein B1 (HMGB1), and peptidoglycan. After activation, TLRs recruit the various downstream molecules, such as myeloid differentiation factor-88 (MyD88), TIR-domain-containing adaptor protein (TIRAP), TNFR-associated factor 6 (TRAF6), and subsequent regulation of activation of NF-*κ*B, leading to the transcription of inflammatory cytokines [107]. It has been confirmed that the TLR pathway is involved in the inflammation response and diseases of CNS. TLR4 knockout inhibits the expression of inflammatory factors IL-1*β* and TNF-*α* and improves the survival rate after induction of intracerebral hemorrhage (ICH) compared with wild-type (WT) mice [108]. TLR4 deficiency suppresses the inflammatory factors IL-1*β* and TNF-*α* and improves cognitive dysfunction in *β*2-microglobulin- (B2M-) induced age-related cognitive decline compared with WT mice [109]. Moreover, several studies have demonstrated that SIRT1 regulates neuroinflammation via inhibition of the TLR pathway. There is a recent study of neuroinflammation induced by excessive ethanol (EtOH), and the study found that the SIRT1 activator resveratrol decreases the inflammatory cytokines IL-1*β* and TNF-*α* and improves the spatial reference memory by inhibition of the TLR2-MyD88-NF-*κ*B signal pathway [110]. Similarly, resveratrol suppresses the proinflammatory cytokines IL-1*β* and TNF-*α*, inhibiting the activation of matrix metalloproteinase-9 (MMP-9) and MMP-2 by downregulation of the TLR-4-NF-*κ*B signaling pathway in trigeminal neuralgia mice [111]. Another study has shown that resveratrol attenuates the cytokines IL-1*β*, IL-6, and TNF-*α* by the TLR4-MyD88-NF-*κ*B pathway in hypoxic-ischemic brain injury mice [112]. An experimental subarachnoid hemorrhage model has shown that resveratrol inhibits the proinflammatory cytokines IL-1*β*, IL-6, and TNF-*α* and ameliorates neurological behavior impairment by the TLR4-MyD88-NF-*κ*B pathway [113].

### 7.3. SIRT1 and NLRP3 Inflammasome

Inflammasomes are cytosolic molecular complexes that sense a variety of stimuli and subsequently trigger and activate inflammatory events to participate in innate immune defenses. Generally, inflammasome complexes consist of a cytosolic sensor (NLR protein or AIM2-like receptor), an apoptosis-associated speck-like protein containing CARD (ASC), and a pro-caspase-1. Once the cytosolic sensor NLR is in response to intracellular signals, this leads to the formation of the inflammasome complex, regulation of activation of caspase-1 and subsequently activated caspase-1 cleavage pro-IL-1*β* and pro-IL-18, and release of the proinflammatory cytokines IL-1*β* and IL-18 [114]. To date, five inflammasome complexes have been found: NLRP1, NLRP3, NLRC4, AIM2, and pyrin inflammasome [115]. In CNS, several cells have been found in the expression of inflammasome components, including microglia, neurons, and astrocytes [116]. Accumulating evidence indicates that activation of the NLRP3 inflammasome is involved in neuroinflammation and neurodegenerative pathologies, and the SIRT1 activator resveratrol can regulate neuroinflammation via inhibition of this pathway. In a cell experiment, resveratrol suppresses the release of inflammatory cytokines IL-1*β*, IL-6, and TNF-*α* in amyloid-beta- (A*β*-) induced BV2 microglial cells by inhibition of the NLRP3 and NF-*κ*B signaling pathways [117]. Similarly, another study found that resveratrol attenuates ATP and LPS-induced NLRP3 inflammasome activation in BV2 microglial cells. The animal experiment of this study also showed that resveratrol suppresses the inflammatory cytokine IL-1*β* and activation of microglia in the hippocampus of the sepsis-associated encephalopathy mice by the NLRP3 signaling pathway [118]. Furthermore, many researchers put their interests in the investigation between SIRT1 activator resveratrol and several animal models of brain injury, including subarachnoid hemorrhage, traumatic brain injury, middle cerebral artery occlusion, and cognitive impairment model. All these studies suggest that the SIRT1 activator resveratrol inhibits the inflammatory cytokines IL-1*β*, IL-18, and TNF-*α* by inhibiting the NLRP3 inflammasome signaling pathway [119–123] (Table 3).

### 7.4. SIRT1 and Mitogen-Activated Protein Kinase

The activation of the mitogen-activated protein kinase (MAPK) pathway in the innate immune response has been widely studied. The stimulation of PRRs on the cell surface can activate the members of MAPK subfamilies, including p38 kinase, Jun N-terminal kinase (JNK), and extracellular signal-regulated kinases (ERK), in conjunction with the NF-*κ*B pathway and many transcription factors of proinflammatory cytokines [124]. It has been found that MAPK pathways contribute to the pathology of several CNS diseases [125]. A growing body of evidence suggests that SIRT1 regulates neuroinflammation involved in MAPK pathways, especially the p38 MAPK pathway. In the study of primary cortical astrocytes, upregulation of SIRT1 by resveratrol or adenoviral vectors inhibits the astrocyte activation after stimulation of IL-1*β* via inhibition of the MAPK signaling pathway [126]. Another study showed that resveratrol suppresses the inflammatory cytokines IL-1*β* and TNF-*α* in hypoxia-induced cytotoxicity in BV2 microglial cells by inhibition of the MAPK and NF-*κ*B signaling pathways [127]. Similarly, resveratrol-enriched rice attenuates several inflammatory cytokines in LPS-activated BV2 microglial cells by suppression of MAPK signaling pathways and NF-*κ*B translocation [128]. There is a study on the neuron cells that found that resveratrol decreases the inflammatory cytokine TNF-*α* and neuron damage in the hippocampus of an alcohol-induced neurodegeneration rat through the p38 MAPK pathway [129]. Moreover, the SIRT1 inhibitor sirtinol promotes the neuronal apoptosis in injured-side cortexes of a traumatic brain injury rat model by the activation of the p38 MAPK pathway [130]. However, there is an opposite conclusion that the SIRT1 inhibitor salermide or SIRT1 siRNA promotes apoptotic neuronal death by inhibition of the ERK1/2 MAPK pathway [131] (Table 4).

## 8. SIRT1 and CNS Diseases

Numerous studies have shown that SIRT1 was implicated in a variety of CNS diseases. The pharmacological SIRT1 activator or inhibitor participant in the regulation of neuroinflammation and neurodegenerative diseases has been reported. Moreover, accumulating studies indicate that synthetic drugs and natural compounds mediate CNS diseases by regulating SIRT1. The following sections discuss the effect of SIRT1 on CNS disorders, including cerebral ischemia, traumatic brain injury, spinal cord injury, AD, and PD.

### 8.1. Cerebral Ischemia

Cerebral ischemia is the most common neurological disease due to the sudden reduction or cessation of blood flow to the brain, which leads to infarction and neuronal dysfunction. Increasing evidence suggests that SIRT1 is a promising target for the treatment of cerebral ischemia injury. In an animal model study, several studies have shown that the SIRT1 activator resveratrol provides a neuroprotective effect in the middle cerebral artery occlusion model [132–136]. One of these studies suggested that SIRT1 exerts the anti-inflammatory and antiapoptotic effects on cerebral ischemic injury by inhibition of acetylation of p53 and NF-*κ*B [130]. Apart from resveratrol, a large number of natural compounds have reported the neuroprotective benefits for cerebral ischemic by activation of SIRT1, while the mechanisms of these compounds that regulate cerebral ischemic are different. The mainly regulatory mechanisms include anti-inflammatory, antiapoptotic, and antioxidative effects [137, 138] (Table 5). For example, rosuvastatin was found to provide a neuroprotective effect against ischemic stroke by inhibiting the NF-*κ*B signaling pathway through the activation of SIRT1 [139]. Salvianolic acid B derived from danshen has a protective effect against ischemic stroke through the activation of SIRT1, leads to an increase of Bcl-2 and a decrease of Bax expression by inhibiting the acetylation of FOXO1, and finally inhibits the apoptosis [140]. Another study has shown that alpha-lipoic acid reduced the ischemic brain damage by the improvement of oxidative damage through the SIRT1/PGC-1*α* pathway [141].

### 8.2. Traumatic Brain Injury

Traumatic brain injury (TBI) is the neurological dysfunction caused by physical force. In addition to this direct insult, secondary injury is driven by a cascade of the inflammatory response, BBB disruption, and metabolic changes [142]. Currently, several studies have demonstrated that the activator of SIRT1 contributes to the improvement of TBI animal models by the involvement of NLRP3 inflammasome and MAPK pathways [122, 126, 143]. Similarly, some studies have shown that an inhibitor of SIRT1 exacerbated the brain damage of TBI models by activation of p38 and ERK1/2 MAPK pathways [130, 131]. Besides, several studies have demonstrated that natural compounds protect against TBI by activation of SIRT1. For example, berberine has reported the neuroprotective effect in a severe TBI mouse model by inhibition of the p38 MAPK pathway through the activation of SIRT1 [144]. Polydatin derived from *Polygonum cuspidatum* ameliorated the damage of TBI through SIRT1-mediated improvement of endoplasmic reticulum stress and mitochondrial injury [145]. Another study has demonstrated that omega-3 polyunsaturated fatty acids (*ω*-3 PUFA) reduced the neuronal apoptosis in TBI rats through the promotion of autophagy by SIRT1-mediated deacetylation of beclin-1 [146]. They also found that *ω*-3 PUFA could inhibit the inflammatory response in the TBI model by SIRT1-mediated deacetylation of HMGB1 and NF-*κ*B [147] (Table 6).

### 8.3. Spinal Cord Injury

Spinal cord injury (SCI) is a devastating neurological disorder which leads not only to motor and sensory deficits but also to a wide range of other organ dysfunctions, such as respiratory and bowel issues, bladder dysfunction, and osteoporosis [148]. Posttraumatic inflammation plays a critical role in the pathogenesis of SCI [149]. Numerous studies have shown that SIRT1 exerts a neuroprotective effect in the animal model of SCI. For instance, SIRT1 agonist CAY10602 inhibited cell apoptosis through the p53 signaling pathway in the SCI rat model [150]. Pretreatment of resveratrol had a neuroprotective effect on spinal cord neuron apoptosis by regulating autophagy via the activation of the SIRT1/AMPK pathway [151, 152]. A study has found that the SIRT1 activator of SRT1720 ameliorated inflammatory response and microglial activation through regulation of the Wnt/*β*-catenin pathway [153]. Consistent with this study, another study had confirmed that SRT1720 suppressed the inflammatory reactions. In addition, SIRT1 knockout mice led to severe motor function, neuronal survival, and inflammatory response than WT mice after SCI [154].

### 8.4. Alzheimer's Disease (AD)

Alzheimer's disease (AD) is the most common neurodegenerative disease that leads to the decline of cognitive function and memory. AD is characterized by the pathological accumulation of amyloid-*β* (A*β*), neurofibrillary tangles (NFTs), and phosphorylated tau protein [155]. In addition, inflammation is thought to exacerbate the pathology of AD [156]. One study has reported that resveratrol provides a beneficial effect on AD rats by downregulation of NF-*κ*B p65, A*β*, and MMP-9 [105]. Another study has shown that resveratrol ameliorated the A*β*-induced cognitive deficits in AD mice by upregulation of SIRT1 and downregulation of NF-*κ*B/IL-1*β*/NLRP3 [157]. However, numerous evidences indicated the beneficial effects of SIRT1 in AD by regulating the expression of A*β* and tau protein. Some research has suggested that resveratrol suppressed the A*β*-induced neuronal apoptosis by activation of SIRT1 pathways [158–160]. Moreover, a recent study has reported that the upregulation of SIRT1 promoted the degradation of A*β* in primary astrocytes [161]. In addition, a study suggested that SIRT1 could suppress the spread of pathogenic tau by deacetylation of tau in tauP301S transgenic mice [162]. Furthermore, accumulating evidence has demonstrated that the SIRT1 agonist resveratrol has a protective effect on multiple animal models of AD [163–168]. For example, the treatment of resveratrol improved learning and memory and suppressed neural apoptosis in the Tg2576 mouse model of AD [163]. Similarly, resveratrol has been found to decrease A*β* levels and improve learning and memory in APP/PS1 AD mice [166]. In addition, another AD model study was induced by A*β*_1–42_, and this study has shown that resveratrol ameliorated the spatial, learning, and memory deficits by regulating SIRT1 signaling pathways [168]. Furthermore, overexpression of SIRT1 in the hippocampus improved learning and memory and reduced the expression of A*β* and tau in the 3xTg AD mouse model [169]. Apart from resveratrol, several natural compounds have reported the beneficial effects of the regulation of SIRT1 in AD animal studies [170–175] (Table 7).

### 8.5. Parkinson's Disease (PD)

Parkinson's disease (PD) is the second most common neurological disorder and is characterized by the loss of dopaminergic (DA) neurons. Aggregation of alpha-synuclein (*α*-syn) of neurons is another pathological feature of PD [176]. Increasing evidence supports the important role of neuroinflammation in the pathogenesis of PD [177]. Accumulating evidence supports the hypothesis that SIRT1 has beneficial effects on different models of PD. Three neurotoxins, including rotenone, 6-hydroxydopamine (6-OHDA), and 1-methyl-4-phenylpyridinium (MPP+), are extensively used in cellular models of PD [178, 179]. Several in vitro studies have found that resveratrol ameliorated neuronal apoptosis in the rotenone-induced SH-SY5Y cell model of PD by regulation of SIRT1 pathways [180, 181]. A recent study has reported that the upregulation of SIRT1 protected SH-SY5Y cells from rotenone by inhibition of NF-*κ*B [182]. In addition, some natural compounds, including epigallocatechin-3-gallate (EGCG), echinacoside (ECH), and salidroside, have reported the neuroprotective effects on MPP+-treated cellular models of PD by regulating SIRT1 pathways [183–185]. Furthermore, some recent studies indicate that the neuroprotective role of SIRT1 in the animal model of PD. For example, activation SIRT1 by resveratrol alleviated the loss of dopaminergic neurons and promoted the degradation of *α*-synuclein by deacetylation of LC3 in the MPTP-induced mouse model [186]. SIRT1 knockout worsens movement function in a mouse PD model induced by MPTP [187]. However, a recent study suggested that SIRT1-transgenic mice fail to alleviate the loss of nigrostriatal dopamine neurons in the MPTP-induced mouse model [188]. A list of studies of treatments for PD that target SIRT1 is shown in Table 8.

## 9. SIRT1 and Brain Aging

Aging is a physiological phenomenon in which the body function declines in a time-dependent fashion. It has been widely reported that brain aging is characterized by the presence of cognitive changes [189]. Aging is a major risk factor for some neurodegenerative diseases, such as AD [190] and PD [191]. Some studies have shown that the inflammatory process and oxidative stress contribute to the cognitive changes in aged brains [192, 193]. Gene expression studies have shown the age-related gene expression changes in the human brain. The downregulation genes which include those related to memory and learning, calcium signaling, and mitochondrial function are downregulated. In addition, the upregulation genes include DNA repair, oxidative stress, and inflammatory response [194–196]. The chronic inflammatory status was shown to link with aging and age-related diseases closely [197]. The major characteristics of the aging process are a progressive increase in proinflammatory status. This phenomenon was firstly called “inflamm-aging” [198, 199]. As discussed in the previous section, SIRT1 plays an important role in the progression of neurodegenerative diseases, such as AD and PD. Increasing evidence shows that SIRT1 is involved in the regulation of aging and lifespan. Brain-specific Sirt1-overexpressing (BRASTO) transgenic mice were shown to have increased lifespans [200], and pharmacologic SIRT1 activator SRT1720 was shown to have lifespan benefits in mice [201]. Growing evidence indicates that SIRT1 can be a connecting link among inflammatory response, oxidative stress, and aging. A recent study has shown that SIRT1-knockout zebrafish resulted in chronic inflammation, oxidative injury, and decreased lifespan [202]. In addition, SIRT1 can regulate oxidative stress, inflammation, and senescence in the progression of COPD [203]. These studies suggest that SIRT1 may be a potential target for aging-associated disorders.

## 10. SIRT1 and Clinical Study

On the basis of the above research, a large body of cellular and animal studies indicates the neuroprotective effect of SIRT1 in neuroinflammation and neurodegenerative diseases. In addition, there are an increasing number of clinical studies to explore the effect of SIRT1 on inflammatory diseases. For example, treating ulcerative colitis subjects with resveratrol for six weeks decreased serum inflammatory markers TNF-*α* and hs-CRP compared to placebo [204]. Another randomized controlled clinical trial demonstrated that treatment with resveratrol for 12 weeks led to the reduction of serum ALT, TNF-*α*, IL-6, and hs-CRP in nonalcoholic fatty liver disease patients [205]. Moreover, there are several clinical trials in the area of CNS diseases. AD subjects who received resveratrol for 52 weeks were able to decrease the expression of cerebrospinal fluid MMP-9 and modulate the inflammatory markers and adaptive immunity [206]. However, another clinical trial of AD indicates that resveratrol has no beneficial effects [207]. In addition, treatment with resveratrol for 26weeks improved memory performance and hippocampal functional connectivity in healthy older adults [208]. In contrast, one recent clinical trial indicates that resveratrol fails to improve verbal memory in older adults [209]. A randomized interventional study showed that resveratrol improved the resting-state functional connectivity (RSFC) of the hippocampus and reduced the serum glycated hemoglobin A1c (HbA1c) in patients with mild cognitive impairment (MCI) [210]. These findings suggest that SIRT1 may be the potential target treatment of neuroinflammation and neurodegenerative disorders. However, in order to explore the therapeutic benefit of SIRT1 on CNS diseases, larger sample clinical trials with longer duration need to be performed in the future.

## 11. Conclusions and Perspectives

In this review, we summarized the latest evidence of the beneficial roles of SIRT1 in the regulation of neuroinflammation and neurodegenerative diseases. As one of the most well-studied sirtuins, SIRT1 can regulate multiple biological functions, including apoptosis, DNA repair, inflammatory response, metabolism, cancer, and stress. We focused on the regulatory effect of SIRT1 in the inflammation response of CNS. Numerous researches indicate that mechanisms of SIRT1 modulate inflammatory reactions in CNS by many different molecules and pathways. Several potential modulatory pathways were summarized in this text, including NF-*κ*B, TLRs, NLRP3 inflammasome, and MAPK pathways. The neuroprotective effects of SIRT1 activation have been reported in in vivo and in vitro models of neuroinflammation-associated diseases. Moreover, some clinical trials have reported the neuroprotective effects of the SIRT1 activator resveratrol. Although a large number of studies demonstrated the benefic effects of the pharmacological activator of SIRT1 in neurological disorders. Some questions still need to be answered. For example, the biological functions of SIRT1 are complex and wide. The precise mechanisms of SIRT1 by deacetylation, which targets the inflammatory process of CNS diseases, are unclear. In addition, the evidence of anti-inflammatory and neuroprotective effects of SIRT1 from clinical studies is not enough. Therefore, further investigations are needed in the precise targets of SIRT1, which will contribute to developing treatment strategies for neuroinflammation-related diseases.

## Figures and Tables

**Table 1:** The substrates of SIRT1.

Substrate	Lysine site	Function	Reference
p53	K382	Apoptosis and senescence	[42, 43]
NF-*κ*B p65	K310	Apoptosis and inflammation	[45]
Ku70	K539, K542, K544, K533, and K556	DNA repair	[48]
FOXO1	K222, K245, K248, K262, K265, K274, and K294	Apoptosis	[50]
FOXO3	K242, K259, K271, K290, and K569	Cell cycle arrest, oxidative stress	[50]
FOXO4	K186, K189, and K408	Oxidative stress	[50]
HIF-1*α*	K674	Cell invasion	[53]
HIF-2*α*	K385, K685, and K741	Cell survival	[53]
PGC-1*α*	K13	Mitochondrial biogenesis and metabolism	[58]
STAT3	K685, K679, K707, and K709	Gluconeogenesis and cell proliferation	[60]

**Table 2:** SIRT1 regulate neuroinflammation involved in the NF-*κ*B pathway.

Chemical agents	Models	Involved mechanisms	Effect	Reference
Resveratrol	Rat astrocytes induced by amyloid-beta (A*β*)	Upregulation of SIRT1 decreases the nuclear translocation of NF-*κ*B p65	Suppression of inflammatory cytokines (TNF-*α*, IL-1*β*, and MCP-1)	Zhao et al. [101]
Resveratrol	N9 microglia cell lines induced by amyloid-beta (A*β*)	Upregulation of SITRT1 decreases the nuclear translocation of NF-*κ*B p65	Suppression of inflammatory cytokines (IL-1*β*, IL-6, and NO)	Zhao et al. [101]
Resveratrol	Primary glial from rat cortices induced by amyloid-beta (A*β*)	Upregulation of SIRT1 inhibits NF-*κ*B p65 by deacetylating Lys310 residue	Suppression of iNOS and EGFP expression; improvement of the survival of MAP2-positive neurons	Chen et al. [102]
Resveratrol	Cerebral ischemia mouse model; primary cortical neurons induced by oxygen-glucose deprivation (OGD)	The high levels of acetylation of NF-*κ*B p65 occur in mouse and cell models; resveratrol deacetylated the Lys310 in primary neurons after OGD	Suppression of LDH expression and improvement of the survival of neuronal cell	Lanzillotta et al. [103]
Resveratrol	Postoperative cognitive dysfunction (POCD) rat model	Upregulation of SIRT1 decreases the expression of acetyl-NF-*κ*B p65 in the hippocampus	Suppression of inflammatory cytokines (TNF-*α*, IL-1*β*, and IL-6)	Yan et al. [73]
Resveratrol	LPS-induced depressive-like behaviors mouse model	Upregulation of SIRT1 decreases the expression of NF-*κ*B in the hippocampus	Suppression of LPS-induced depression-like behaviors and the overactivation of microglia	Liu et al. [104]
Resveratrol	Alzheimer's disease rat model induced by ovariectomized (OVX)+D-galactose (D-gal)	Upregulation of SITRT1 decreases the expression of NF-*κ*B p65 in the hippocampus	Suppression of insoluble amyloid-beta (A*β*) and MMP-9; increase of the tight junction protein claudin-5	Zhao et al. [105]

**Table 3:** SIRT1 regulate neuroinflammation involved in the NLRP3 inflammasome pathway.

Chemical agents	Models	Involved mechanisms	Effect	Reference
Resveratrol	BV2 microglia cell lines induced by amyloid-beta (A*β*)	Upregulation of SIRT1 inhibits TXNIP/TRX/NLRP3 and NF-*κ*B signaling pathway	Suppression of inflammatory cytokines (IL-1*β*, IL-6, and TNF-*α*)	Feng and Zhang [117]
Resveratrol	BV2 microglia cell lines induced by ATP and LPS	Upregulation of SIRT1 inhibits NLRP3 inflammasome signaling pathway	Suppression of inflammatory cytokine IL-1*β*	Sui et al. [118]
Resveratrol	Sepsis-associated encephalopathy mouse model induced by cecal ligation and puncture (CLP)	Upregulation of SIRT1 inhibits NLRP3 inflammasome signaling pathway	Suppression of inflammatory cytokine IL-1*β*, activation of microglia in the hippocampus	Sui et al. [118]
Resveratrol	Estrogen deficiency-induced depression-like behavior mouse model	Upregulation of SIRT1 inhibits NLRP3 inflammasome and NF-*κ*B signaling pathway	Suppression of inflammatory cytokines (IL-1*β* and IL-18) and activation of microglia in the hippocampus	Liu et al. [119]
Resveratrol	Subarachnoid hemorrhage rat model	Upregulation of SIRT1 inhibits NLRP3 inflammasome signaling pathway	Suppression of inflammatory cytokines (IL-1*β*, IL-18, and TNF-*α*), microglia activation, and neutrophil infiltration	Zhang et al. [120]
Resveratrol	Isoflurane-induced cognitive impairment mouse model	Upregulation of SIRT1 inhibits the expression of NLRP3 inflammasome	Suppression of inflammatory cytokines ( IL-1*β* and TNF-*α*) in the hippocampus	Li et al. [121]
Resveratrol	Traumatic brain injury rat model	Upregulation of SIRT1 inhibits NLRP3 inflammasome signaling pathway	Suppression of inflammatory cytokines ( IL-1*β* and IL-18) and ROS	Zou et al. [122]
Resveratrol	Middle cerebral artery occlusion (MCAO) rat model	Resveratrol inhibits NLRP3 inflammasome signaling pathway by upregulated autophagy	Suppression of inflammatory cytokines (IL-1*β* and IL-18)	He et al. [123]

**Table 4:** SIRT1 regulate neuroinflammation involved in the MAPK pathway.

Chemical agents	Models	Involved mechanisms	Effect	Reference
Resveratrol or adenoviral vectors	IL-1*β*-stimulated primary cortical astrocyte model	Overexpression of SIRT1 or resveratrol inhibits the MAPK pathway	Suppression of the astrocyte activation	Li et al. [126]
Resveratrol	Hypoxia-induced cytotoxicity in BV2 microglial cells	Resveratrol inhibits ERK and JNK MAPK signaling pathways and the NF-*κ*B pathway	Suppression of inflammatory cytokines (IL-1*β* and TNF-*α*)	Zhang et al. [127]
Resveratrol-enriched rice (RR)	LPS-activated BV2 microglial cells	RR inhibits MAPK signaling pathways and NF-*κ*B translocation	Suppression of inflammatory cytokines (iNOS, COX-2, TNF-*α*, IL-1*β*, and IL-6)	Subedi et al. [128]
Resveratrol	Alcohol-induced neurodegeneration rat and SH-SY5Y cells	Resveratrol inhibits the activation of the p38 MAPK pathway	Suppression of inflammatory cytokine TNF-*α* and reduction of alcohol-induced neuron damage in the hippocampus	Gu et al. [129]
Sirtinol	Traumatic brain injury (TBI) rat model	Sirtinol exacerbate the activation of the p38 MAPK pathway	Promotion of TBI-induced mitochondrial damage and neuronal apoptosis in injured-side cortexes	Yang et al. [130]
Salermide and SIRT1 siRNA	Primary cortical neurons induced by scratch injury	Salermide and SIRT1 siRNA inhibit the activation of the ERK1/2 pathway	Promotion of apoptotic neuron death	Zhao et al. [131]
Salermide	Traumatic brain injury (TBI) mice model	Salermide inhibits the activation of the ERK1/2 pathway	Promotion of apoptotic neuron death in injured-side cortex	Zhao et al. [131]

**Table 5:** Synthetic drugs and natural compounds regulate ischemic stroke by involving in SIRT1 in animal models.

Chemical agents	Models	Involved pathway	Effect	Reference
Resveratrol	Transient MCAO mice	SIRT1-Akt/ERK/p38/PGC-1*α*	Antioxidative, antiapoptotic, and anti-inflammatory effects	Shin et al. [132]
Activator 3, sirtinol	pMCAO mice	SIRT1/p53/NF-*κ*B p65	Neuroprotective effect	Hernández-Jiménez et al. [133]
Resveratrol	MCAO mice	SIRT1-Akt/HO-1	Antiapoptotic effects	Hermann et al. [134]
Resveratrol	MCAO mice	SIRT1-BDNF	Neuroprotective effect	Koronowski et al. [135]
Resveratrol	MCAO rats	cAMP/AMPK/SIRT1	Neuroprotective effect	Wan et al. [136]
Magnolol	MCAO rats	SIRT1/FOXO1/Bcl-2/Bax	Anti-inflammatory and antiapoptotic effects	Kou et al. [137]
Nampt	MCAO rats	Nampt/SIRT1/AMPK	Neuroprotective effect	Wang et al. [138]
PEA-OXA	MCAO rats	SIRT1/NF-*κ*B/Bcl-2/Bax	Anti-inflammatory and antiapoptotic effects	Fusco et al. [211]
Liraglutide	MCAO mice	SIRT1/ICDH/*α*-KGDH/SDH	Improvement of mitochondrial enzyme activity	He et al. [212]
Rosuvastatin	Cerebral ischemic stroke rat model	SIRT1/NF-*κ*B p65	Neuroprotective effect	Yan and Zhu [139]
Salvianolic acid B	MCAO rats	SIRT1/FOXO1/Bcl-2/Bax	Anti-inflammatory and antiapoptotic effects	Lv et al. [140]
Alpha-lipoic acid	pMCAO rats	SIRT1/PGC-1*α*	Antioxidative effect and improvement of neurological deficit and brain edema	Fu et al. [141]
Arctigenin	MCAO rats	SIRT1/NLRP3 inflammasome	Anti-inflammatory effects	Zhang et al. [213]
CDP-choline	MCAO rats	SIRT1	Neuroprotective effect	Hurtado et al. [214]
Curcumin	MCAO rats	SIRT1/p53/Bcl-2/Bax	Anti-inflammatory effects	Miao et al. [215]
Icariin	MCAO mice	SIRT1/PGC-1*α*	Improvement of neurological scores and brain edema	Zhu et al. [216]

pMCAO: permanent middle cerebral artery occlusion; PEA-OXA: N-palmitoylethanolamide-oxazoline.

**Table 6:** Synthetic drugs and natural compounds regulate traumatic brain injury by involving in SIRT1 in animal models.

Chemical agents	Models	Involved pathway	Effect	Reference
Resveratrol	TBI rats	SIRT1/NLRP3 inflammasome	Anti-inflammatory effects	Zou et al. [122]
Resveratrol	TBI mice	SIRT1/MAPK	Suppression of the astrocyte activation	Li et al. [126]
Resveratrol	TBI mice	SIRT1/autophagy	Neuroprotective effect	Zhang et al. [143]
Sirtinol	TBI rats	SIRT1/p38	Exacerbations of neuronal damage	Yang et al. [130]
Salermide	TBI mice	SIRT1/ERK1/2	Promotion of apoptotic neuron	Zhao et al. [131]
Berberine	TBI mice	SIRT1/p38	Neuroprotective effect	Wang and Zhang [144]
Polydatin	TBI rats	SIRT1/p38/p-PERK/XBP-1/ATF6	Antiapoptotic effects	Li et al. [145]
*ω*-3 PUFA	TBI rats	SIRT1/beclin-1	Antiapoptotic effects	Chen et al. [146]
*ω*-3 PUFA	TBI rats	SIRT1/HMGB1/NF-*κ*B	Anti-inflammatory effects	Chen et al. [147]

**Table 7:** Synthetic drugs and natural compounds regulate Alzheimer's disease by involving in SIRT1 in animal models.

Chemical agents	Models	Involved pathway	Effect	Reference
Resveratrol	OVX- and D-gal- induced AD rat model	SIRT1/NF-*κ*B p65	Suppression of A*β* and MMP-9	Zhao et al. [105]
Resveratrol	AD mouse model induced by A*β*1–42	SIRT1/AMPK/PGC-1*α*/NLRP3/NF-*κ*B	Improvement of cognitive deficits	Qi et al. [157]
Resveratrol	Tg2576 mouse AD model	SIRT1	Improvement of learning and memory, suppression of neural apoptosis	Wang et al. [163]
Resveratrol	Diabetes and AD rat model	SIRT1	Improvement of memory deficits	Ma et al. [164]
Resveratrol	APP/PS1 AD mice	SIRT1	Decrease of senile plaques and antioxidant effects	Dong et al. [165]
Resveratrol	APP/PS1 AD mice	SIRT1/ERK1/2	Improvement of learning and memory and decrease of A*β*	Cao et al. [166]
Resveratrol	APP/PS1 AD mouse model	SIRT1/AMPK	Improvement of memory loss and decrease of amyloid burden	Porquet et al. [167]
Resveratrol	AD rat model induced by A*β*1–42	SIRT1/CREB	Improvement of spatial, learning, and memory	Wang et al. [168]
—	3xTg AD mouse model	SIRT1/BDNF	SIRT1-transgenic mice improve cognitive behavior and decrease A*β* and tau pathology	Corpas et al. [169]
SLAB51	3xTg AD mouse model	SIRT1	Antioxidant effects	Bonfili et al. [170]
24-OH	hTau mice	SIRT1	Decrease of hyperphosphorylated tau protein	Testa et al. [171]
Acetylshikonin	D-galactose-induced AD mouse model	SIRT1/p53/p21	Improvement of cognitive impairment and hippocampus senescence	Li et al. [172]
Melatonin	A*β*-induced AD mouse model	SIRT1/TFAM	Improvement of memory and hippocampal cell damage	Ansari Dezfouli et al. [173]
Dihydromyricetin	A*β*-induced AD rat model	AMPK/SIRT1	Improvement of cognitive function and suppression of inflammatory responses and cell apoptosis	Sun et al. [174]
Salidroside	D-gal-induced AD rat model	SIRT1/NF-*κ*B	Suppression of inflammatory responses	Gao et al. [175]

D-gal: D-galactose; OVX: ovariectomized; A*β*: amyloid-*β*; APP: amyloid precursor protein; PS1: presenilin 1.

**Table 8:** Synthetic drugs and natural compounds regulate Parkinson's disease by involving in SIRT1 in cell and animal models.

Chemical agents	Models	Involved pathway	Effect	Reference
Resveratrol	6-OHDA-treated SH-SY5Y cells	SIRT1/BMAL1	Antioxidant effects	Wang et al. [179]
Resveratrol	Rotenone-induced SH-SY5Y cells	SIRT1/p53	Suppression of neural apoptosis	Feng et al. [180]
Resveratrol	Rotenone-induced SH-SY5Y cells	AMPK/SIRT1/autophagy	Suppression of neural apoptosis and promotion of the degradation of *α*-synuclein	Wu et al. [181]
Oxyresveratrol	6-OHDA-treated SH-SY5Y cells	SIRT1/JNK	Neuroprotective effects	Chao et al. [217]
DCHC	6-OHDA-treated PC-12 cells	SIRT1	Suppression of neural apoptosis	Tsai et al. [218]
SIRT1 viral plasmid	Rotenone-treated SH-SY5Y cells	SIRT1/NF-*κ*B	Increase of cell survival and decrease of *α*-synuclein aggregates	Singh et al. [182]
EGCG	MPP+-treated PC12 cells	SIRT1/PGC-1*α*	Suppression of oxidative stress	Ye et al. [183]
ECH	MPP+-treated PC12 cells	SIRT1/FOXO1/autophagy	Increase of cell survival	Chen et al. [184]
Salidroside	MPP+-treated SH-SY5Y cells	SIRT1/MAPK	Suppression of neural apoptosis and oxidative stress	Wang et al. [185]
Resveratrol	MPTP-induced mouse model	SIRT1/PGC-1*α*	Neuroprotective effects	Mudò et al. [219]
Resveratrol	MPTP-induced mouse model	SIRT1/LC3	Protect the loss of dopaminergic neurons and promote the degradation of *α*-synuclein	Guo et al. [186]
—	MPTP-induced mouse model	SIRT1	SIRT1 knockout mice worsen movement function	Zhang et al. [187]
—	MPTP-induced mouse model	SIRT1	SIRT1-transgenic mice fail to alleviate loss of nigrostriatal dopamine neurons	Kitao et al. [188]

MPP+: 1-methyl-4-phenyl-pyridine; DCHC: 3-(2,4-dichlorophenyl)-7-hydroxy-4H-chromen-4-one; EGCG: epigallocatechin-3-gallate; 6-OHDA: 6-hydroxydopamine; ECH: echinacoside; MPTP: 1-methyl-4-phenyl-1,2,3,6-tetrahydropyridine.

## Data Availability

The data used to support the findings of the study can be available from the corresponding author.
